# Development and evaluation of machine learning models based on X-ray radiomics for the classification and differentiation of malignant and benign bone tumors

**DOI:** 10.1007/s00330-022-08764-w

**Published:** 2022-04-09

**Authors:** Claudio E. von Schacky, Nikolas J. Wilhelm, Valerie S. Schäfer, Yannik Leonhardt, Matthias Jung, Pia M. Jungmann, Maximilian F. Russe, Sarah C. Foreman, Felix G. Gassert, Florian T. Gassert, Benedikt J. Schwaiger, Carolin Mogler, Carolin Knebel, Ruediger von Eisenhart-Rothe, Marcus R. Makowski, Klaus Woertler, Rainer Burgkart, Alexandra S. Gersing

**Affiliations:** 1 Department of Radiology, Klinikum rechts der Isar, School of Medicine, Technische Universität München, Ismaninger Strasse 22, 81675 Munich, Germany; 2 Department for Orthopedics and Orthopedic Sports Medicine, Ismaninger Strasse 22, 81675 Munich, Germany; 3 Department of Diagnostic and Interventional Radiology, Medical Center–University of Freiburg, Faculty of Medicine, Freiburg, Germany; 4 Department of Neuroradiology, Klinikum rechts der Isar, School of Medicine, Technische Universität München, Munich, Germany; 5 Institute of Pathology, Klinikum rechts der Isar, School of Medicine, Technische Universität München, Ismaninger Strasse 22, 81675 Munich, Germany; 6 Department of Neuroradiology, University Hospital, LMU Munich, Marchioninistraße 15, 81377 Munich, Germany

**Keywords:** Bone neoplasms, Musculoskeletal system, Diagnostic imaging, Radiography, Machine learning

## Abstract

**Objectives:**

To develop and validate machine learning models to distinguish between benign and malignant bone lesions and compare the performance to radiologists.

**Methods:**

In 880 patients (age 33.1 ± 19.4 years, 395 women) diagnosed with malignant (*n* = 213, 24.2%) or benign (*n* = 667, 75.8%) primary bone tumors, preoperative radiographs were obtained, and the diagnosis was established using histopathology. Data was split 70%/15%/15% for training, validation, and internal testing. Additionally, 96 patients from another institution were obtained for external testing. Machine learning models were developed and validated using radiomic features and demographic information. The performance of each model was evaluated on the test sets for accuracy, area under the curve (AUC) from receiver operating characteristics, sensitivity, and specificity. For comparison, the external test set was evaluated by two radiology residents and two radiologists who specialized in musculoskeletal tumor imaging.

**Results:**

The best machine learning model was based on an artificial neural network (ANN) combining both radiomic and demographic information achieving 80% and 75% accuracy at 75% and 90% sensitivity with 0.79 and 0.90 AUC on the internal and external test set, respectively. In comparison, the radiology residents achieved 71% and 65% accuracy at 61% and 35% sensitivity while the radiologists specialized in musculoskeletal tumor imaging achieved an 84% and 83% accuracy at 90% and 81% sensitivity, respectively.

**Conclusions:**

An ANN combining radiomic features and demographic information showed the best performance in distinguishing between benign and malignant bone lesions. The model showed lower accuracy compared to specialized radiologists, while accuracy was higher or similar compared to residents.

**Key Points:**

*• The developed machine learning model could differentiate benign from malignant bone tumors* *using radiography with an AUC of 0.90 on the external test set.*

*• Machine learning models that used radiomic features or demographic information alone performed worse than those that used both radiomic features and demographic information as input, highlighting the importance of building comprehensive machine learning models.*

*• An artificial neural network that combined both radiomic and demographic information achieved the best performance and its performance was compared to radiology readers on an external test set.*

**Supplementary Information:**

The online version contains supplementary material available at 10.1007/s00330-022-08764-w.

## Introduction

Conventional radiography is considered to be the initial imaging modality of choice for the diagnostics of bone tumors and tumor-like lesions [1–3]. Radiography allows accurate visualization of osseous destruction patterns and of periosteal response patterns [2]. This enables an assessment of the biological activity of bone lesions and with which lesions can be categorized into aggressive or non-aggressive bone lesions [4]. Further features, such as matrix mineralization or the tumor architecture, may additionally help to establish a specific diagnosis and thus make conventional radiographs crucial for the diagnostic work-up of bone tumors and tumor-like lesions and the following therapy [5]. Magnetic resonance imaging may help with narrowing differential or when a lesion is indeterminate by demonstrating extraosseous tissue components or the composition of the tumor. However, radiography is considered the primary imaging method of choice because it visualizes certain features of bone lesions (e.g. periosteal reaction, osseous destruction pattern, matrix mineralization) combined with high resolution, cost-effectiveness, and accessibility[3, 6].

To standardize the assessment of bone lesions on radiographs, methods enabling the computer-aided extraction of imaging features can be used. For this purpose, radiomics have been successfully used to distinguish between benign and malignant lesions [7–9]. Radiomics make use of the extraction of a multitude of imaging features to deduct an image-based signature that characterizes a tumor [10]. The radiomic signatures can then be used as input for machine learning models to classify the tumor [11]. Machine learning models include statistical models, decision tree models, support vector machines, and artificial neural networks (ANN) [10]. Decision trees such as random forest classifiers (RFC) or statistical methods such as logistic regression or Gaussian Naive Bayes classifiers (GNB) are widely used for classification tasks [12]. A previous study demonstrated the use of radiographic imaging features and demographic information to build a GNB model for the classification of bone tumors [13]. Therefore, we hypothesized that combining radiomics extracted from radiographs with demographic information may allow for reliable characterization of bone lesions. This may be particularly helpful for the clinical diagnostic routine, since the assessment of certain bone lesions requires expertise in musculoskeletal tumor imaging, which is difficult to acquire outside of a specialized center, due to the rarity with which these occur.

The aim of this proof-of-concept study was therefore to develop and validate machine learning models using radiomics derived from radiographs and demographic information to distinguish between benign and malignant bone lesions on radiographs and compare the performance to radiologists on an external test set.

## Materials and methods

### Patient selection and dataset

The local institutional review boards approved this retrospective multi-center study (Technical University Munich and University of Freiburg). The study was performed in accordance with national (as specified in Drs. 7301-18) and international guidelines (as specified in European Medicines Agency guidelines for good clinical practice E6). Informed consent was waived for this retrospective anonymized study. Radiographs of all eligible patients with primary bone tumors obtained at the primary institution between January 1, 2000, and December 31, 2019, were selected for this study, forming a consecutive series. The imaging protocols were in accordance with those previously described [1]. Patients included in this study (*n* = 880, average age 33.1 years ± 19.4, 395 women) were diagnosed by histopathology which was considered to be the standard of reference with malignant tumors (chondrosarcoma, *n* = 87; osteosarcoma, *n* = 34; Ewing’s sarcoma, *n* = 32; plasma cell myeloma, *n* = 28; B cell non-Hodgkin’s (NHL) lymphoma, *n* = 36; chordoma, *n* = 6) and benign tumors (osteochondroma, *n* = 228; enchondroma, *n* = 153; chondroblastoma, *n* = 19; osteoid osteoma, *n* = 19; giant cell tumor of bone, *n* = 44; Non-ossifying fibroma, *n* = 34; hemangioma, *n* = 12; aneurysmal bone cyst, *n =* 82; simple bone cyst, *n* = 24; fibrous dysplasia, *n* = 52). Chondrosarcomas included atypical cartilaginous tumors (grade 1, *n* = 8), grade 2 (*n* = 48), and grade 3 (*n* = 31) chondrosarcomas. Patients were excluded (*n* = 51) due to poor image quality because of artifacts not allowing for any analysis or radiologic assessment of the tumor. Metastases were excluded as they were not included in the database of the university’s musculoskeletal tumor center as primary bone tumors. All included cases were reviewed by two radiologists independently (A.S.G., musculoskeletal fellowship-trained radiologist with 8 years of experience, and S.C.F. a radiologist with 4 years of experience) to ensure that the tumor was correctly depicted on the radiograph with sufficient quality to locate and segment the bone tumor. The dataset was split randomly into 70%/15%/15% for training, validation, and internal testing.

Additionally, an external test set comprised of 96 patients from a different institution (Freiburg University Hospital) was used for further independent testing. Likewise, the external cohort was selected through the database of another university’s musculoskeletal tumor center forming a consecutive series.

Since an increasing model performance with a higher number of training cases was expected all eligible patients with primary bone tumors from the main institution were included to obtain as many samples for the development data set as possible. A sample size of 80–100 cases for the external validation was included, similar to comparable research studies [14, 15].

Table 1 gives an overview of the patients included in this study and tumor types.

**Table 1:** Subject characteristics*

Subject characteristics	Overall (*n* = 880)	Training set (614/887, 70%)	Validation set (133/887, 15%)	Test set (133/887, 15%)	External Test Set (*n* = 96)
**Age** (years ± SD)	33.1 ± 19.4	34.0 ± 19.9	31.8 ± 17.3	30.3 ± 18.5	31.7 ± 22.1
**Sex** (females)	395 (44.9%)	275 (44.8%)	60 (45.1%)	60 (45.1%)	40 (41.7%)
**Malignant subtypes**	213 (24.2%)	149 (24.3%)	32 (24.1%)	32 (24.1%)	31 (32.3%)
Chondrosarcoma	87 (9.8%)	61 (9.9%)	13 (9.8%)	13 (9.8%)	11 (11.5%)
Osteosarcoma	34 (3.8%)	24 (3.9%)	5 (3.8%)	5 (3.8%)	7 (7.3%)
Ewing’s sarcoma	32 (3.6%)	22 (3.6%)	5 (3.8%)	5 (3.8%)	5 (5.2%)
Plasma cell myeloma	28 (3.2%)	20 (3.3%)	4 (3.0%)	4 (3.0%)	4 (4.2%)
NHL B cell	26 (2.9%)	18 (2.9%)	4 (3.0%)	4 (3.0%)	4 (4.2%)
Chordoma	6 (0.6%)	4 (0.6%)	1 (0.7%)	1 (0.7%)	0 (0%)
**Benign subtypes**	667 (75.8%)	465 (75.7%)	101 (75.9%)	101 (75.9%)	65 (67.7%)
Osteochondroma	228 (25.9%)	160 (26.1%)	34 (25.6%)	34 (25.6%)	16 (16.7%)
Enchondroma	153 (17.4%)	107 (17.4%)	23 (17.3%)	23 (17.3%)	12 (12.5%)
Chondroblastoma	19 (0.2%)	13 (2.1%)	3 (2.3%)	3 (2.3%)	(2.1%)
Osteoid osteoma	19 (0.2%)	13 (2.1%)	3 (2.3%)	3 (2.3%)	1 (1.0%)
Giant cell tumor of bone	44 (4.7%)	30 (4.6%)	7 (5.0%)	7 (5.0%)	6 (6.2%)
Non-ossifying fibroma	34 (3.9%)	24 (3.9%)	5 (3.8%)	5 (3.8%)	7 (7.3%)
Haemangioma	12 (1.4%)	8 (1.3%)	2 (1.5%)	2 (1.5%)	3 (3.1%)
Aneurysmal bone cyst	82 (9.3%)	58 (9.4%)	12 (9.0%)	12 (9.0%)	8 (8.3%)
Simple bone cyst	24 (2.7%)	16 (2.6%)	4 (3.0%)	4 (3.0%)	5 (5.2%)
Fibrous dysplasia	52 (5.9%)	36 (5.9%)	8 (6.0%)	8 (6.0%)	5 (5.2%)
**Location**					
Torso/head	118 (13.4%)	79 (12.9%)	16 (12.0%)	23 (17.3%)	16 (16.7%)
Upper extremity	234 (26.6%)	166 (27.0%)	28 (21.1%)	40 (30.0%)	29 (30.2%)
Lower extremity	528 (60.0%)	369 (60.1%)	89 (66.9%)	70 (52.6%)	51 (53.1%)

*Data is given as mean ± standard deviation; data in parentheses are percentages. The internal data set was split for training, validation, and testing 70%, 15%, 15%, respectively. The external test set obtained from a different institution was included for further independent testing. Malignant tumors included chondrosarcoma, osteosarcoma, Ewing’s sarcoma, chordoma, plasma cell myeloma, and b cell non-Hodgkin’s lymphoma NHL. Benign tumors included osteochondroma, enchondroma, chondroblastoma, osteoid osteoma, non-ossifying fibroma NOF, giant cell tumor, haemangioma, simple and aneurysmatic bone cyst, and fibrous dysplasia

The imaging data was extracted from Digital Imaging and Communications in Medicine (DICOM) files as portable network graphics (PNG) files. PNG was chosen to ensure further lossless image processing. Segmentations of the tumors were performed blinded to the histopathological and clinical data by one radiologist (S.C.F.), using the open-source software (3D Slicer, version 4.7; www.slicer.org) and reviewed by A.S.G. [16]. To measure the intrareader reliability of the tumor segmentations, 45 patients were randomly selected from the data set and an additional segmentation was performed 3 months after the initial segmentation. The recorded intrareader reliability as measured by dice score was 0.92 ± 0.13.

To compare the performance to radiologists, two radiology residents (C.E.v.S., Y.L.) and two radiologists specialized in musculoskeletal tumor imaging (A.S.G., P.J.M.) classified the radiographs of the external test set.

### Radiomic feature extraction and machine learning model development

Image processing, feature extraction, machine learning model development, and validation were performed on a 16-core Intel-i9 9900K CPU at 3.60 GHz (Intel), 32GB-DDR4-SDRAM running Linux system (Ubuntu 18.04 Canonical) and implemented in Python 3.7.7 (open-source, Python Software Foundation). Radiomic features were extracted as defined in the pyRadiomics library (version 3.0, https://www.radiomics.io/pyradiomics.html) [7]. The image of the DICOM file was extracted as PNG for further preprocessing. The extracted features are then used as input for the ML models. Clinical information such as location of the tumor (torso/head, upper extremity, lower extremity), age, and sex was also used as input.

**Fig. 1:**
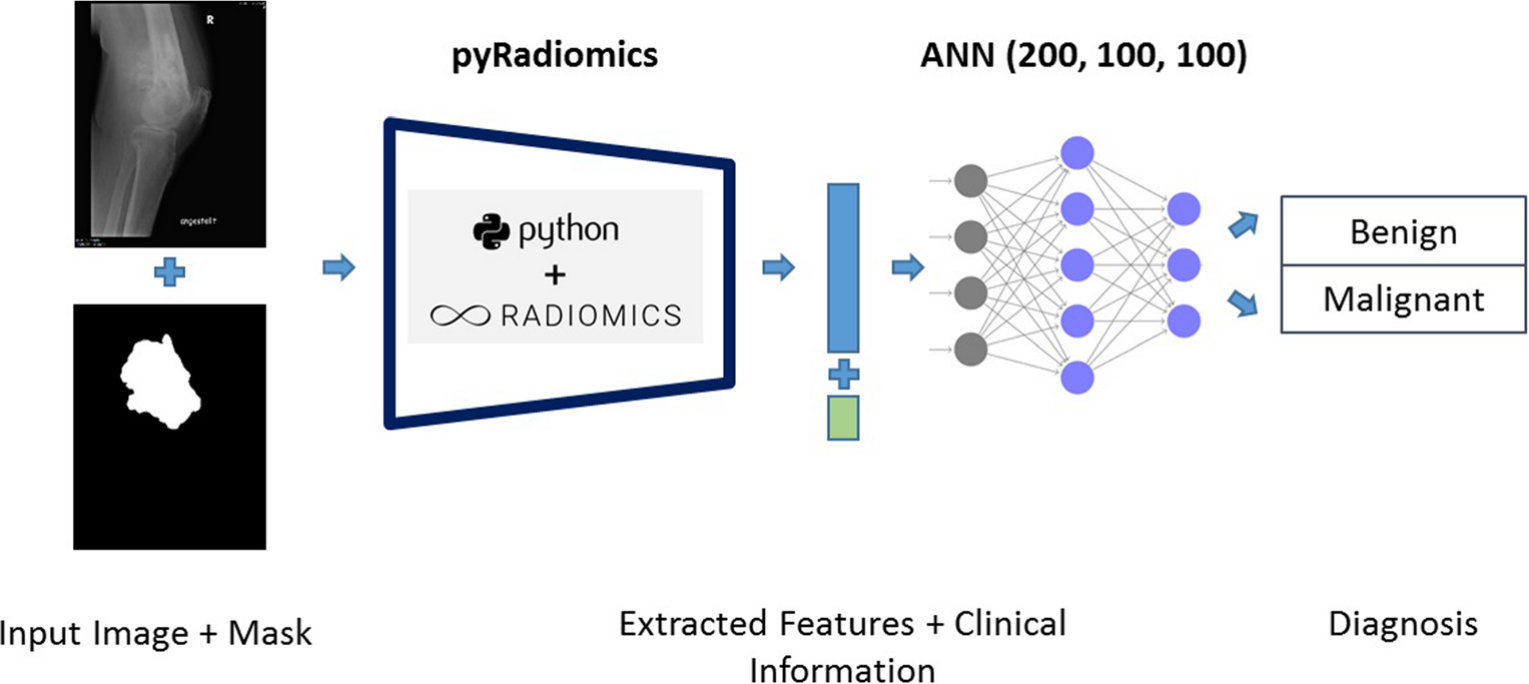
Overview of the utilized pipeline. The image and binary mask are fed to the pyRadiomics model to extract all relevant radiomic features. The extracted features and clinical information are then sent to an ANN in order to distinguish between benign and malignant tumors

First, a RFC was trained using 200 estimators and a maximum depth of 3 as defined previously [17]. This model enabled a detailed analysis of the relevant radiomic features, which motivated the classification. The 10 most important features were selected from the RFC model. Additionally, a GNB and an ANN with 3 fully connected layers and 200, 100, and 100 neurons in each layer were trained using the scikit-learn 0.22.2 (scikit-learn.org) and fastai library [18]. Figure 1 shows an overview of image processing and analysis steps that allow radiomic analysis and machine learning model development. The exact description of the machine learning methods with the model and training parameters used can be found as code online (https://github.com/NikonPic/bonetumor-radiomics).

### Model evaluation and statistical analysis

Machine learning models were developed on the training set and validated on the validation set. The best-performing models on the validation set were chosen for final evaluation on the test sets. The model performance reported in this study was observed on the test sets using confusion matrices, accuracy, positive and negative predictive value, precision, recall, f1-score, receiver-operating characteristics (ROC) with area under the curve (AUC) analysis, and 95% confidence intervals (CI) with Clopper-Pearson’s method using scikit-learn 0.22.2 (scikit-learn.org) as previously defined [19]. Sensitivity was calculated as the number of true positives (correctly identified malignant bone tumors) divided by the number of true positives (correctly identified malignant bone tumors) and number of false negatives (malignant bone tumors incorrectly classified as benign). Specificity was calculated as the number of true negatives (correctly identified as benign bone tumors) divided by the number of true negatives (correctly identified as benign bone tumors) and the number of false positives (benign bone tumors incorrectly classified as malignant). McNemar’s test was used for statistical comparison and *p* < 0.05 was assumed to be statistically significant. Assuming a difference in the accuracy of 7.5% between the model and the resident as well as the musculoskeletal radiologist with a desired level of confidence of 95% using McNemar’s test resulted in at least 80 as the sample size of a test set. Additionally, the softmax of the output of the ANN was calculated as an estimate for the certainty of the prediction. Standard deviations and confidence intervals of the AUCs were calculated with pROC (1.16.1) using the DeLong method in R (3.6.1) [20]. Statistical analyses were performed by B.J.S. Model training, evaluation, and visualization were performed by C.E.v.S. (8 years of experience in data analysis) and N.J.W. (computer scientist with 8 years of experience in statistics and data analysis).

## Results

### Radiomic feature evaluation and demographic information

**Fig. 2:**
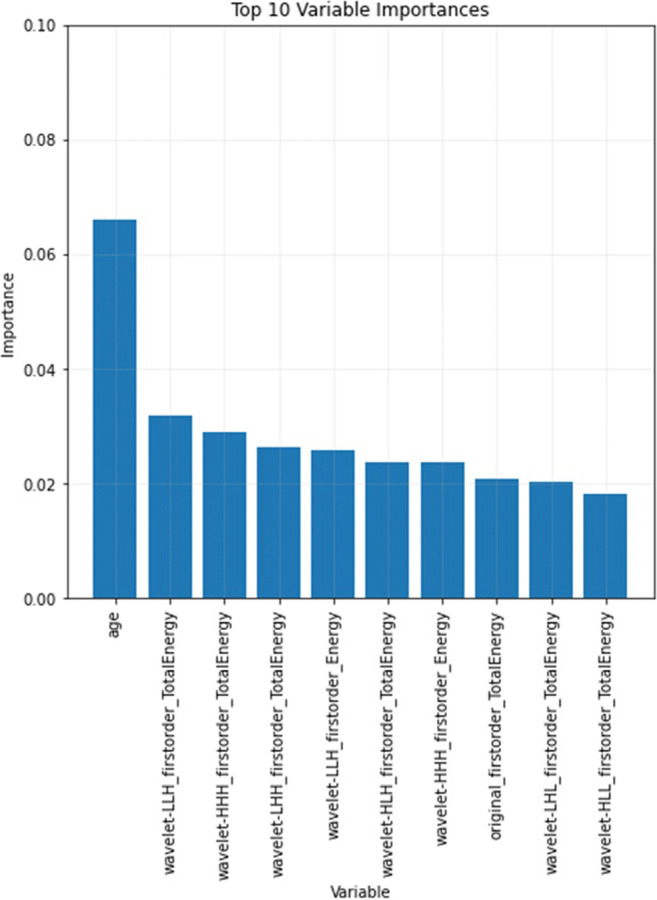
Visualization of the 10 most important features with their relative importance of the random forest classifier

Overall, more than 200 radiomic features were analyzed. Of all radiomic features and demographic information, ‘age’ and ‘LLH_firstorder_TotalEnergy’ showed the highest relevance according to their feature importance with the RFC as also demonstrated in Fig. 2. To further investigate the discriminatory power of individual features, ANNs were trained for 10 epochs each. Only a single of these ten most relevant features was used as input. These models showed moderate classification performance with AUCs from 0.49 to 0.65 or accuracies from 52 to 62% depending on the feature that was used, highlighting that using a single radiomic feature or a single demographic variable was not sufficient to accurately distinguish benign from malignant primary bone tumors but rather a combination of radiomic and/or demographic information was needed. Interestingly, of the extracted radiomic features, those that focused on the intensity of individual and neighboring pixels, as well as those that reflected inhomogeneity, were more relevant. Detailed information on the individual radiomic feature performance can be found in Table 2.

**Table 2:** Performance on the 10 most significant radiomic and demographic features alone

Feature	AUC	Accuracy	Sensitivity	Specificity
age	0.49 ± 0.01	0.58 ± 0.01	0.33 ± 0.09	0.67 ± 0.05
wavelet-LLH_firstorder_TotalEnergy	0.64 ± 0.01	0.6 ± 0.05	0.73 ± 0.03	0.55 ± 0.08
wavelet-HHH_firstorder_TotalEnergy	0.65 ± 0.02	0.61 ± 0.03	0.71 ± 0.04	0.57 ± 0.05
wavelet-LHH_firstorder_TotalEnergy	0.63 ± 0.01	0.62 ± 0.01	0.71 ± 0.04	0.59 ± 0.03
wavelet-LLH_firstorder_Energy	0.61 ± 0.01	0.58 ± 0.02	0.56 ± 0.05	0.59 ± 0.05
wavelet-HLH_firstorder_TotalEnergy	0.65 ± 0.01	0.55 ± 0.03	0.76 ± 0.03	0.48 ± 0.05
wavelet-HHH_firstorder_Energy	0.6 ± 0.01	0.59 ± 0.03	0.56 ± 0.1	0.59 ± 0.07
original_firstorder_TotalEnergy	0.59 ± 0.02	0.52 ± 0.02	0.78 ± 0.03	0.42 ± 0.03
wavelet-LHL_firstorder_TotalEnergy	0.6 ± 0.01	0.55 ± 0.01	0.67 ± 0.03	0.51 ± 0.03
wavelet-HLL_firstorder_TotalEnergy	0.57 ± 0.02	0.52 ± 0.02	0.72 ± 0.03	0.45 ± 0.04

*Data is given as mean ± standard deviation

### Machine learning model evaluation of combined radiomics and demographic information

Using the available demographic information, the best performing model was a RFC with 0.75 AUC, 76% accuracy (101/133, 95% CI: 0.68, 0.83), 41% sensitivity (13/32, 95% CI: 0.24, 0.59) and 87% specificity (88/101, 95% CI: 0.79, 0.93). In comparison, using the selected radiomic features, the best performing model was an ANN with an 0.71 AUC, 75% accuracy (100/133, 95% CI: 0.67, 0.82), 66% sensitivity (21/32; 95% CI: 0.47, 0.81), and 78% specificity (79/101; 95% CI: 0.69, 0.86).

Combining radiomic features and demographic information as input to an ANN resulted in a remarkable increase in performance with 0.79 AUC, 80% accuracy (107/133, 95% CI: 0.73, 0.87), 75% sensitivity (24/32, 95% CI: 0.57, 0.89), and 82% specificity (83/101, 95% CI: 0.73, 0.89) as well as a positive and negative predictive value of 57% (24/42, 95% CI: 0.41, 0.72) and 91% (83/91, 95% CI: 0.83, 0.96), respectively. This model achieved higher accuracy than the model based on demographic information or radiomic features alone and demonstrated an increase in accuracy by 4% and 5% (*p* = 0.041 and *p* = 0.023), respectively.

Table 3 shows the classification performances of all developed models. RFC, GNB, and ANN were used as architectures. For each architecture, models were developed that used radiomic features only, demographic information only, or a combination of both radiomic features and demographic information.

**Table 3:** The classification performances of the models on the internal test set using radiomic features or demographic information alone, as well as combining both radiomic features and demographic information. As model architectures, the following three were used: A random forest classifier (RFC), a Gaussian naïve Bayes classifier (GNB), and an artificial neural network (ANN)*

Model architecture	Score	Demographic features	Radiomic features	Combined: radiomic + demographic features
**RFC (200 estimators)**	**AUC**	0.75	0.73	0.76
	**Accuracy**	0.76 (101/133; 95% CI: 0.68, 0.83)	0.59 (78/133; 95% CI: 0.50, 0.67)	0.60 (80/133; 95% CI: 0.51, 0.69)
	**Sensitivity**	0.41 (13/32; 95% CI: 0.24, 0.59)	0.84 (27/32; 95% CI: 0.67, 0.95)	0.81 (26/32; 95% CI: 0.64, 0.93)
	**Specificity**	0.87 (88/101; 95% CI: 0.79, 0.93)	0.5 (51/101; 95% CI: 0.40, 0.61)	0.53 (54/101; 95% CI: 0.43, 0.63)
**GNB**	**AUC**	0.72	0.68	0.68
	**Accuracy**	0.44 (59/133; 95% CI: 0.36, 0.53)	0.76 (101/133; 95% CI: 0.68, 0.83)	0.76 (101/133; 95% CI: 0.68, 0.83)
	**Sensitivity**	0.92 (29/32; 95% CI: 0.75, 0.98)	0.44 (14/32; 95% CI: 0.26, 0.62)	0.44 (14/32; 95% CI: 0.26, 0.62)
	**Specificity**	0.29 (29/101; 95% CI: 0.20, 0.39)	0.86 (87/101; 95% CI: 0.78, 0.92)	0.86 (87/101; 95% CI: 0.78, 0.92)
**ANN (200, 100, 100)**	**AUC**	0.59	0.71	0.79
	**Accuracy**	0.67 (89/133; 95% CI: 0.58, 0.75)	0.75 (100/133; 95% CI: 0.67, 0.82)	0.80 (107/133; 95% CI: 0.73, 0.87)
	**Sensitivity**	0.38 (12/32; 95% CI: 0.21, 0.56)	0.66 (21/32; 95% CI: 0.47, 0.81)	0.75 (24/32; 95% CI: 0.57, 0.89)
	**Specificity**	0.76 (77/101; 95% CI: 0.67, 0.84)	0.78 (79/101; 95% CI: 0.69, 0.86)	0.82 (83/101; 95% CI: 0.73, 0.89)

*In parenthesis proportions are given as numerical values and 95% confidence intervals (CI) are provided. Area under the curve (AUC) were obtained from receiver operating characteristics (ROC)

### Machine learning model evaluation on the external test set and comparison with radiologists

On the external test set, the best performing ANN achieved an AUC of 0.90, an accuracy of 75% (72/96, 95%: 0.65, 0.83), a sensitivity of 90% (28/31, 95% CI: 0.74, 0.98), and a specificity of 68% (44/65, 95% CI: 0.55, 0.79) as well as a positive and negative predictive value of 57% (28/49, 95% CI: 0.42, 0.71) and 94% (44/47, 95% CI: 0.82, 0.99), respectively.

The first radiology resident achieved 72% accuracy (68/96, 95% CI: 0.61, 0.80), 61% sensitivity (19/31, 95% CI: 0.42, 0.78), and 75% specificity (49/65, 95% CI: 0.63, 0.85). In comparison, the model showed similar accuracy (*p* = 0.134) at significantly better sensitivity (*p* < 0.01) and similar specificity (*p* = 0.074).

The second radiology resident achieved 65% accuracy (62/96, 95% CI: 0.54, 0.74), 35% sensitivity (11/31, 95% CI: 0.19, 0.55), and 78% specificity (51/65, 95% CI: 0.67, 0.88). In comparison, the model showed higher accuracy (*p* < 0.01) with better sensitivity (*p* < 0.01) at lower specificity (*p* = 0.023).

The first radiologist specialized in musculoskeletal tumor imaging achieved 84% accuracy (81/96, 95% CI: 0.76, 0.91), 90% sensitivity (28/31, 95% CI: 0.74, 0.98), and 82% specificity (53/65, 95% CI: 0.70, 0.90). In comparison, the model showed lower accuracy (*p* < 0.01) at similar sensitivity (*p* = 1) and lower specificity (*p* < 0.01).

The second radiologist specialized in musculoskeletal tumor imaging achieved 83% accuracy (80/96, 95% CI: 0.74, 0.90), 81% sensitivity (25/31, 95% CI: 0.63, 0.93), and 85% specificity (55/65, 95% CI: 0.74, 0.92). In comparison, the model showed lower accuracy (*p* = 0.013) at similar sensitivity (*p* = 0.248) and lower specificity (*p* < 0.01).

The prevalence of malignant bone tumors was higher in the external test set compared to the internal test set, possibly leading to differences in the performance measures of the ANN between the internal and external test set.

**Fig. 3:**
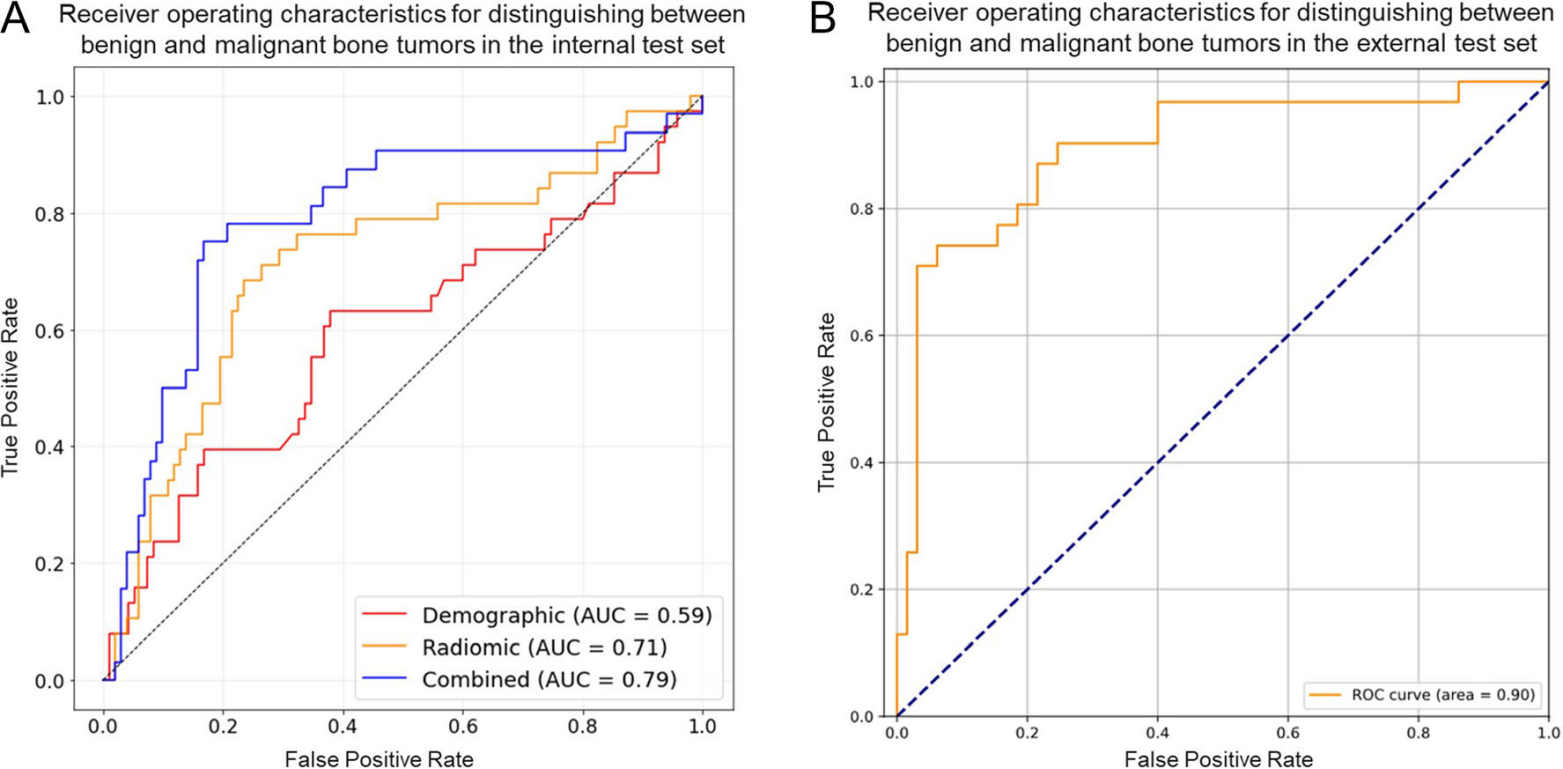
**A** shows the receiver operating characteristics (ROC) on the internal test set for three artificial neural networks (ANN). One ANN was based on demographic information alone (red). Another ANN was based on radiomic features alone (yellow). A third ANN was based on the combination of demographic information and radiomic features (blue). The ANN based on both demographic information and radiomic features displayed the highest discriminatory power. **B** shows the ROC on the external test set for the ANN combining demographic and radiomic features

**Fig. 4:**
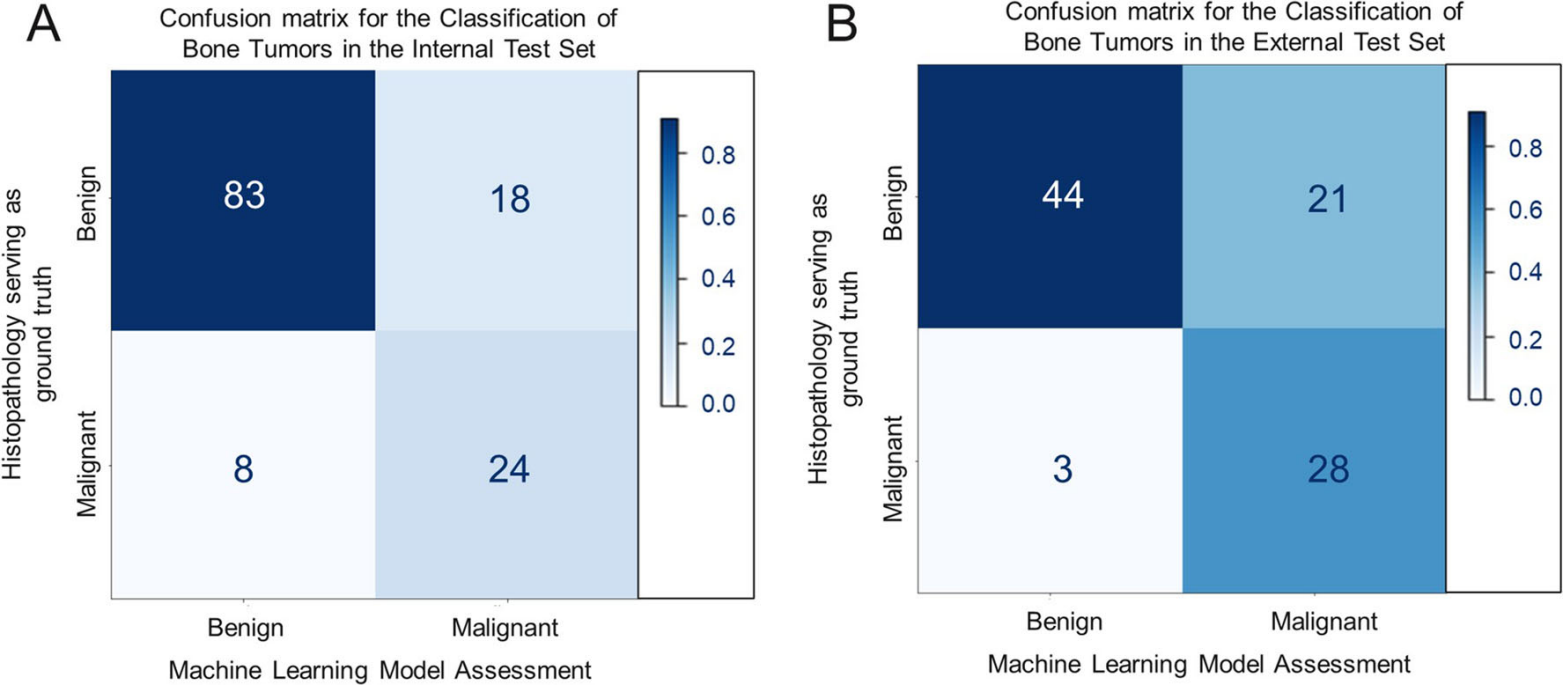
**A** shows a confusion matrix of the overall best performing model, an artificial neural network (ANN) combing both radiomic and demographic information on the internal test set. **B** shows the confusion matrix of the same model on the external test set obtained from another institution for further, independent testing

Figure 3A shows the ROC on the internal test set for the best performing model, an ANN using both radiomic features and demographic information, as well as the ANNs based on demographic information or radiomic features alone. Figure 3B shows the performance of the best-performing model on the external test set. Figure 4A and B show the confusion matrices for the best-performing model — an ANN combining both radiomic and demographic information on the internal and external test set, respectively.

### Examples of correct and incorrect classifications by the best performing model

**Fig. 5:**
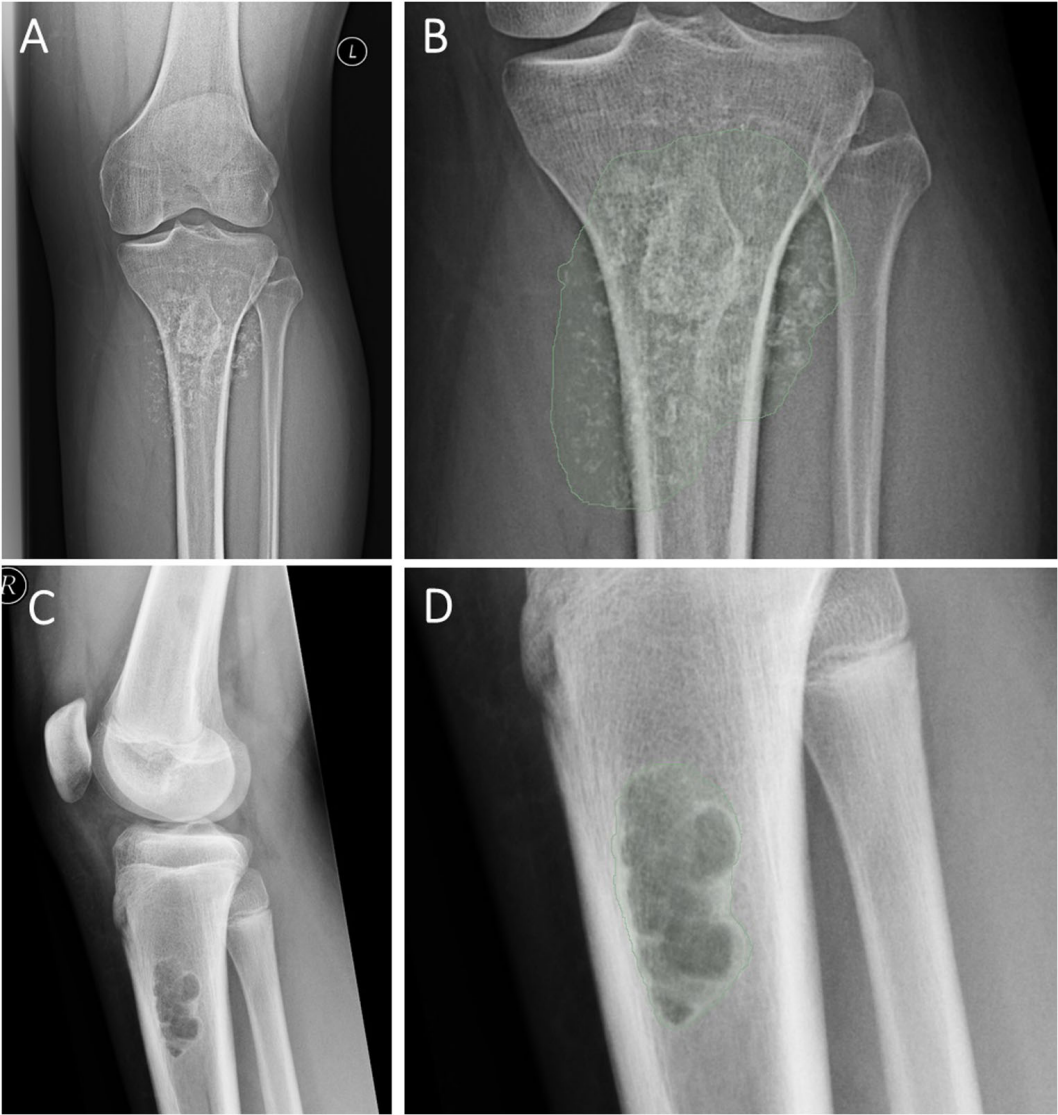
**A** and **B** Example of a malignant tumor in the tibia of a 33-year-old male with a chondrosarcoma. **A** shows the radiograph and **B** shows the segmentation for the radiomics extraction. The artificial neural network model combining both demographic and radiomic information correctly predicted a malignant tumor with a certainty of 86%. **C** and **D** Example of a benign tumor in the proximal tibia of a 15-year-old male with a non-ossifying fibroma. **A** shows the radiograph and **B** shows the segmentation for the radiomics extraction. The artificial neural network model using the combination of both, the demographic and radiomic information, correctly predicted a benign tumor with a certainty of 93%

**Fig. 6:**
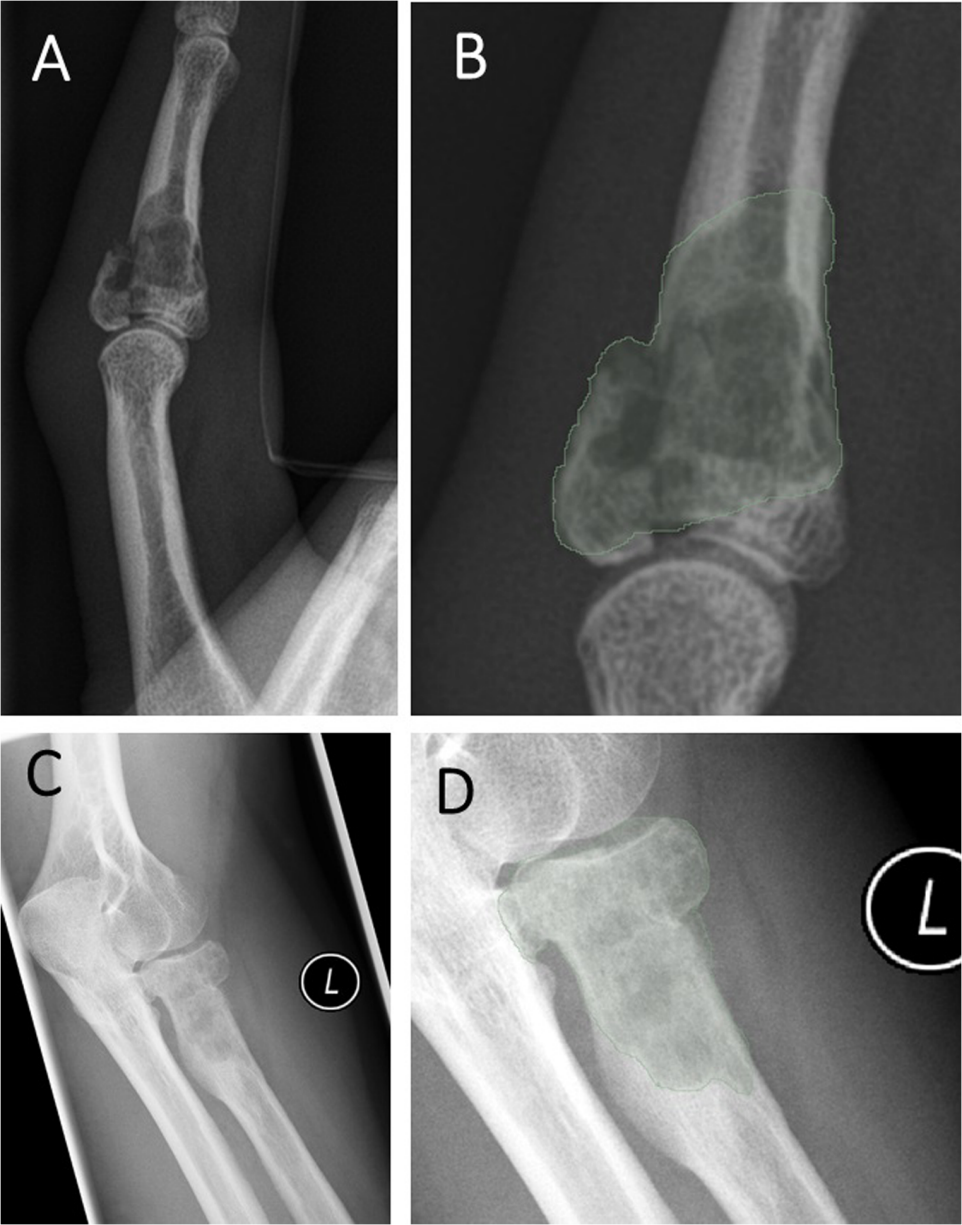
**A** and **B** Example of a misclassified tumor from a 41-year-old female with an enchondroma and a pathological fracture through the tumor. **A** shows the radiograph and **B** shows the segmentation for the radiomics extraction. The artificial neural network model combining both demographic and radiomic information incorrectly classified this tumor as malignant with a certainty of 54%. **C** and **D** Example of a malignant tumor from a 45-year-old diagnosed with a chondrosarcoma. **A** shows the radiograph and **B** shows the segmentation for the radiomics extraction. The artificial neural network model combining both demographic and radiomic information correctly predicted a malignant tumor with a certainty of 67%

Cases of correct and incorrect classifications by the best performing ANN combining both radiomic features and demographic information were reviewed to further investigate the functioning of the model. When reviewing correct classifications, we could identify cases that showed patterns of malignancy as demonstrated in Fig. 5 A and B or showed the typical appearance of benign lesions as shown in Fig. 5C and D. Those cases also showed high certainty of prediction of the ANN with 86% and 93% certainty, respectively. Figure 6A and B show a case of a benign tumor that was misclassified as a malignant tumor with a low prediction certainty of 54%. This may have occurred due to the pathological fracture of the benign tumor. Figure 6C and D show a case of correct classification of a malignant tumor with a low to moderate prediction certainty of 67%.

## Discussion

In this study, machine learning models based on radiomics and demographic information were developed and validated to distinguish between benign and malignant bone lesions on radiographs and compared to radiologists on an external test set. Overall, machine learning models using the combination of radiomics and demographic information showed a higher diagnostic accuracy than machine learning models using radiomics or demographic information only. The best model was based on an ANN that used both radiomics and demographic information. On an external test set, this model demonstrated lower accuracy compared to radiologists specialized in musculoskeletal tumor imaging, while accuracy was higher or similar compared to radiology residents.

Interestingly, when evaluating individual radiomic features only, features that reflect large differences in densities of neighboring pixels and inhomogeneity showed the highest discriminatory power indicating malignancy. This is in line with other studies assessing the ability of radiomic features based on magnetic resonance imaging, computed tomography (CT), and positron emission tomography (PET)-CT in order to distinguish between benign and malignant lesions in different types of diseases as it may be a reflection of moth-eaten appearance or a very inhomogeneous destruction pattern and may therefore be more often detected in malignant bone tumors compared with benign bone tumors.[9, 21, 22]. Of the evaluated demographic features, age showed the highest discriminatory power, which is in accordance with previous studies [1, 20].

Moreover, previous studies used a combination of radiographic features and demographic information to assess bone tumors on radiographs [13, 23, 24]. Kahn et al used Bayesian networks to differentiate among 5 benign and 5 malignant lesions achieving 68% accuracy [24]. Bao H. Do et al used a naive Bayesian model to differentiate primary and secondary bone tumors using 710 cases achieving 62% primary accuracy to differentiate between 10 distinct diagnoses [13]. Yet, these approaches used radiologist-defined semantic features only assessed on radiographs as input for the models, thus depending on the quality of the readings of the radiologists. In contrast, in this study, radiomic features containing first-, second-, and higher-order statistics were combined with patient data as input for sophisticated machine learning models [10]. Additionally, it needs to be noted that the sample sizes of all of the above mentioned previous studies on radiographic feature assessment of bone tumors were smaller than in this study, and performances were not evaluated on a separate hold-out test set or an external test set, in contrast to current best practices as performed in this study [3, 25, 26].

Due to the varying settings in which patients with bone lesions present, a quantitative method for image analysis may guarantee the highest quality of bone tumor diagnostics in the shortest time. Therefore, automated quantitative evaluation techniques of conventional radiographs obtained during the clinical routine diagnostic workup in patients with bone lesions are needed since these are independent of the experience level in evaluating conventional radiographs of the treating physicians. In this proof-of-concept study, we were able to develop a machine learning model using both radiomic features extracted from radiographs and demographic information with an accuracy higher or similar compared to radiology residents. Therefore, a model such as this implemented into the clinical routine pipeline may support inexperienced or moderately experienced radiologists or physicians in enhancing the quality of their decision-making regarding their diagnosis and consequently the further management or referral of these patients. More specifically, a model such as this may help with ‘ruling-in’ malignant lesions, particularly when the treating physician or the radiologist has limited experience. The patient could then be referred to a specialized center and a biopsy may be performed to secure the diagnosis.

This study has limitations. First, radiomic analysis of the tumor was only performed on a single radiograph without considering more available projections. Second, the applied technique relied on manual segmentations of the bone tumor. However, automated segmentations of bone tumors on radiographs may be developed in the future. Third, the ANN is limited by the information entailed in a radiograph and may be improved with additional information obtained from magnetic resonance imaging. Fourth, the demographic information included the location of the tumor, age, and sex; however, the medical history and clinical symptoms such as pain level and duration were also important and their use may be explored in future studies. Also, the developed models can currently only differentiate between benign and malignant lesions and not between the different tumor subtypes. However, a multitude of bone tumors, particularly malignant tumors, cannot be differentiated further by radiography alone, as indicated by the low accuracies in the previous studies mentioned above. In particular, some x-ray features of benign and malignant bone tumors may overlap, such as in low-grade chondrosarcoma showing a benign growth pattern or in giant cell tumor of bone sometimes demonstrating an aggressive growth pattern and periosteal reaction. Moreover, the dataset included only patients with histopathological diagnoses of the osseous lesions, since histopathology was considered to be the standard of reference in our study. Therefore, this may have created a selection bias that we cannot account for, since from certain bone lesions such as NOFs or fibrous dysplasia, histopathology is usually only obtained under circumstances in which bone stability seems endangered or the lesion itself appears to be ‘atypical’. Finally, bone metastases were not included in the current study, while they make up a large part of the malignant bone lesions.

This study is therefore considered to be a proof-of-concept study and the developed machine learning models need to be tested, optimized, and further evaluated in larger datasets also including bone metastases and additionally consisting of conventional radiographs with the final diagnosis of bone lesions based on the clinical and imaging consensus as well as on histopathology in future studies.

In conclusion, a machine learning model using both radiomic features and demographic information was developed that showed high accuracy and discriminatory power for the distinction between benign and malignant bone tumors on radiographs of patients that underwent biopsy. The best model was based on an ANN that used both radiomics and demographic information resulting in an accuracy higher or similar compared to radiology residents. A model such as this may enhance diagnostic decision-making especially for radiologists or physicians with limited experience and may therefore improve the diagnostic work up of bone tumors.

## Supplementary information

ESM 1 (DOCX 27 kb)

## Abbreviations

ANN: Artificial neural network
AUC: Area under the curve
CI: Confidence interval
DICOM: Digital Imaging and Communications in Medicine
GNB: Gaussian naive Bayes Classifier
NHL: Non-Hodgkin’s lymphoma
NOF: Non-ossifying fibroma
PNG: Portable network graphics
RFC: Random forest classifier

## Declarations

### Guarantor

The scientific guarantor of this publication is A.S.G.

### Statistics and biometry

One of the authors has significant statistical expertise (B.J.S.).

### Methodology

- retrospective
- diagnostic or prognostic study
- multicentre study
