# Supplementary material for: Development and evaluation of machine learning models based on X-ray radiomics for the classification and differentiation of malignant and benign bone tumors
Source: Eur Radiol. 2022 Apr 9;32(9):6247–57. doi: 10.1007/s00330-022-08764-w (PMC9381439; doi:10.1007/s00330-022-08764-w)
Supplement: Supplementary file 1 — (DOCX 27 kb) [file 330_2022_8764_MOESM1_ESM.docx]

**Supplementary Material Development and Evaluation of Machine Learning Models based on X-Ray Radiomics for the Classification and Differentiation of Malignant and Benign Bone Tumors**

**Supplementary Table 1:** Performance of the artificial neural network (ANN) combing both radiomic and demographic information on the external test set in comparison to the two radiology residents and the two radiologists specialized in musculoskeletal tumor imaging with 95% confidence intervals (95% CI).

| **External**  **Test Set** | **Artificial  Neural Network** | **Radiology Resident 1** | **Radiology Resident 2** | **Specialized Radiologist 1** | **Specialized Radiologist 2** |
| --- | --- | --- | --- | --- | --- |
| ***Accuracy***  ***(95% CI)*** | **0.75**  **(0.65, 0.83)** | **0.71**  **(0.61, 0.80)** | **0.65**  **(0.54, 0.74)** | **0.84**  **(0.76, 0.91)** | **0.83**  **(0.74, 0.90)** |
| ***Sensitivity***  ***(95% CI)*** | **0.90**  **(0.74, 0.98)** | **0.61**  **(0.42, 0.78)** | **0.35**  **(0.19, 0.55)** | **0.90**  **(0.74, 0.98)** | **0.81**  **(0.63, 0.93)** |
| ***Specificity***  ***(95% CI)*** | **0.68**  **(0.55, 0.79)** | **0.75**  **(0.63, 0.85)** | **0.78**  **(0.67, 0.88)** | **0.82**  **(0.70, 0.90)** | **0.85**  **(0.74, 0.92)** |
